# Study on the Microstructure and Properties of CoCrFeNiMo High-Entropy Alloy Coatings Prepared by Atmospheric Plasma Spraying

**DOI:** 10.3390/nano15221692

**Published:** 2025-11-08

**Authors:** Chunxia Jiang, Wenge Li, Ziyan Li, Lu Wang, Rongbin Li, Yanlong Xu, Tao Jiang, Yuantao Zhao

**Affiliations:** 1 Merchant Marine College, Shanghai Maritime University, Shanghai 201306, China; jiangcx@sdju.edu.cn (C.J.); lyxu@shmtu.edu.cn (Y.X.); zhaoyt@shmtu.edu.cn (Y.Z.); 2 School of Materials Science and Engineering, Shanghai Dianji University, Shanghai 201306, China; 24600005130201@st.sdju.edu.cn (Z.L.); 231002460220@st.sdju.edu.cn (L.W.); 32187@sdju.edu.cn (T.J.)

**Keywords:** CoCrFeNiMo high-entropy alloy coatings, plasma spraying, spraying current, microstructure, microhardness, tribology

## Abstract

This study employed atmospheric plasma spraying (APS) technology to successfully fabricate CoCrFeNiMo high-entropy alloy (HEA) coatings under varying spraying currents and systematically investigated the effects of the spraying current on the microstructure, mechanical properties, and tribological behavior of the coatings. Results showed that the material composition remained consistent across different current levels, primarily consisting of face-centered cubic (FCC) solid solution phases, FeCr_2_O_4_ spinel phases, and Cr-rich FCC1 phases. The FCC matrix was dispersed with spherical Cr oxide particles smaller than 30 nm in diameter, which significantly enhanced the strength of the coatings. As spraying current increased, both porosity and microhardness exhibited a non-monotonic trend—initial optimization followed by deterioration. At 500 A spraying current, the coating achieved optimal performance, with the lowest porosity (0.42%) and highest microhardness (569.8 HV). Correspondingly, this condition also yielded the best wear resistance, with stable friction coefficients and wear rates reaching 0.49 and 6.91 × 10^−5^ mm^3^/N m, respectively. Abrasion surface analysis revealed that excessively low or high currents triggered distinct wear mechanisms leading to reduced wear resistance.

## 1. Introduction

In harsh service environments like aerospace, energy facilities, and marine engineering, the friction and wear failure of materials significantly limits the service life of mechanical components [1,2,3,4,5,6]. The stability of traditional materials is significantly limited in environments characterized by high temperature and high corrosion. HEAs are multi-component alloys composed of five or more metals in equimolar or near-equimolar ratios [7,8,9]. Their high-entropy effect suppresses the formation of intermetallic compounds while promoting simple solid solution structures like FCC or BCC. Compared to conventional alloys, HEAs exhibit exceptional properties because of their four major effects [10]: ultra-high strength [11,12,13], outstanding corrosion resistance [14,15], high-temperature stability [16,17], and good low-temperature toughness [18]. The typical CoCrFeNi HEAs [19,20,21] have shown great development potential in the industrial field.

However, bulk HEA materials have high processing costs and complex processing techniques, making it difficult for them to be widely used in the industrial field. HEA coatings prepared by techniques like magnetron sputtering, laser cladding, thermal spraying, and electrochemical deposition [22,23,24] can significantly improve the performance of substrate materials. Furthermore, by controlling process parameters and composition during coating preparation, the microstructures and performance of these coatings can be further optimized. In recent years, researchers have extensively studied the friction and wear properties of HEA coatings. Bih-Show Lou [25] prepared VNbMoTaWCr_x_ coatings with different Cr contents using direct current magnetron sputtering and carried out dry friction tests, friction tests in 3.5 wt% sodium chloride aqueous solution, and tribocorrosion tests under applied potential. The results showed that the corrosion resistance and wear resistance of the coatings increased with the increase in Cr content. The FeCrCoNiMo_0.5_W_X_ [26] coatings prepared by laser cladding technology had an FCC phase, σ phase, and μ phase. The study showed that the addition of W element reduced the lattice parameter of the coating, increased the grain size, and intensified the lattice distortion. Due to the synergistic effect of second-phase strengthening, fine-grain strengthening, and solid-solution strengthening, the friction and wear properties of the coating were greatly improved.

APS technology is a widely used thermal spraying technique [27]. This process involves converting gases such as Ar, He, and N_2_ into plasma, which is then discharged from a nozzle to generate an ultra-high-temperature, high-velocity plasma jet serving as the heat source. The central temperature of the plasma arc is usually around 2 × 10^4^ K, making it widely applicable in the preparation of high-melting-point metal coatings and ceramic coatings [28]. Owing to the high velocity of the plasma jet, the thermal spraying material collides with the substrate at a high speed, endowing the coating with high adhesion strength and high density [29]. In recent years, there have also been some reports on the preparation of HEA coatings using APS technology. The CoCrFeNi-X [30,31,32] wear-resistant coatings prepared by APS have become a research focus in recent years.

Currently, most studies on the properties of HEA coatings focus on obtaining coatings with favorable performance by adjusting process parameters. However, they lack in-depth research on the intrinsic relationship among process parameters, coating microstructure, and macroscopic properties. This deficiency leaves the optimization of processes without theoretical guidance, making it difficult to achieve the directional design of coating performance. Based on the above reasons, this paper prepared CoCrFeNiMo high-entropy alloy coatings using APS and systematically investigated the effects of different spraying currents on the coating’s microstructural characteristics, including its microstructure, phase composition, and porosity. Additionally, the influence of the microstructure on the macroscopic properties of the coatings, such as microhardness and wear resistance, was analyzed. By conducting an in-depth analysis of the coating’s wear mechanism, this study provides theoretical support and a practical basis for the development and practical application of high-entropy alloy wear-resistant coatings. The results of this study will offer a theoretical basis for the development of HEA wear-resistant coatings.

## 2. Materials and Methods

**Figure 1:**
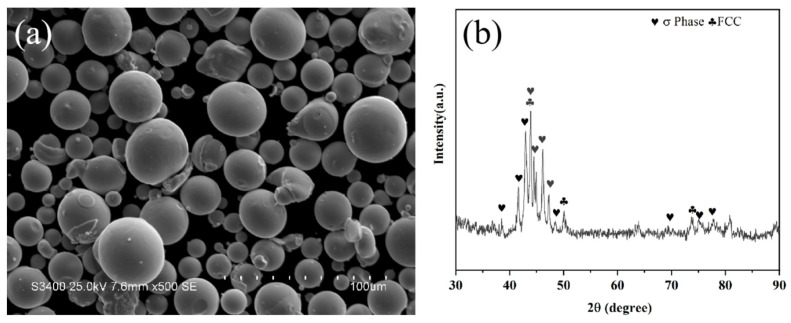
Morphology (**a**) and XRD pattern (**b**) of CoCrFeNiMo powders.

The substrate used in the experiment was a Q235 steel plate, with dimensions of 90 mm × 50 mm × 5 mm. The subsequent test samples were cut into small metal blocks of 10 mm × 10 mm × 5 mm. The morphology and XRD pattern of the powder are shown in Figure 1. The spraying powder was CoCrFeNiMo high-entropy alloy powder, with a particle size ranging from 15 μm to 53 μm. The CoCrFeNiMo powder waws mainly composed of the FCC phase and the intermetallic compound sigma (σ) phase (CrFe_2.32_MoNi). The substrate surface was cleaned with absolute ethanol and acetone to remove surface oil and grease. Then, the substrate was subjected to sandblasting treatment; after treatment, it was cleaned again with absolute ethanol to remove surface sandblasting dust. The spraying process was completed in an atmospheric environment using the Multicoat System plasma spraying system manufactured by Oerlikon Metco (Wohlen, Switzerland). The specific spraying parameters were as follows: spraying currents were set to 300 A, 400 A, 500 A, 600 A, and 700 A; the distance between the spray gun and the substrate was 90 mm; the spray step size was 2 mm; the spray moving speed was 1000 mm/s; and the powder feed rate was 40 g/min. For the gases used in spraying, the argon flow rate was 40 L/min, the hydrogen flow rate was 8 L/min, and the nitrogen flow rate was 3.2 L/min. Each sample was sprayed with 10 coating layers.

The microstructure and morphology of the CoCrFeNiMo coatings were characterized using various instruments, including X-ray diffraction (XRD), white light interferometry, scanning electron microscopy (SEM), energy dispersive spectroscopy (EDS), and transmission electron microscopy (TEM). The phase structure analysis of the coating was conducted using an XRD (Rigaku Ultima IV, Tokyo, Japan) equipped with Cu Kα radiation. During the test, the operating voltage and current were set to 40 kV and 30 mA, respectively; the scanning step size was 0.02°, the scanning speed was 5°/min, and the test angle range was 30–90°. The morphological analysis of the coating was performed using a HITACHI S-3400N tungsten filament (Hitachi, Tokyo, Japan) SEM equipped with EDS. The accelerating voltage of the SEM was set to 20 kV. For the determination of coating porosity, the morphological characteristics were observed at 10 random positions under a magnification of 500×. The images were processed using ImageJ software (ImageJ 1.54i), and the average porosity of the coatings was calculated accordingly. The atomic-scale microstructure analysis of the coatings was performed using a TEM (FEI Tecnai G2 F30 analytical, Hitachi Company, Tokyo, Japan). The sample was cut into thin slices of 10 mm × 10 mm × 0.5 mm by wire cutting. After cleaning, the slices were thinned to 50 μm by mechanical thinning, and then the TEM samples were prepared by ion thinning under a vacuum environment. Diffraction images were obtained in the selected area electron diffraction mode, and the spots of the electron diffraction patterns were calibrated to analyze the crystal structure.

The microhardness test of the coatings was conducted using an HXD-1000 digital microhardness tester(Shanghai Optical Instrument Factory, Shanghai, China). The test load was set to 200 g, and the load holding time was 15 s. For each sample, tests were performed at 10 different positions, and the average value was taken as the final hardness value of the sample. The friction and wear performance test was carried out using a GF-1 reciprocating friction and wear tester (Lanzhou Zhongke Kaihua Technology Development Co., Ltd., Lanzhou, China). The test parameters were as follows: test load of 20 N, friction radius of 5 mm, rotational speed of 300 rpm, and the counter-abrasive ball was GCr15 (a type of high-carbon chromium bearing steel) with a diameter of 5 mm; the friction time was 30 min. After the test, a white light interferometer was used to characterize the wear scars, and the corresponding wear rate was calculated.

## 3. Results and Discussion

**Figure 2:**
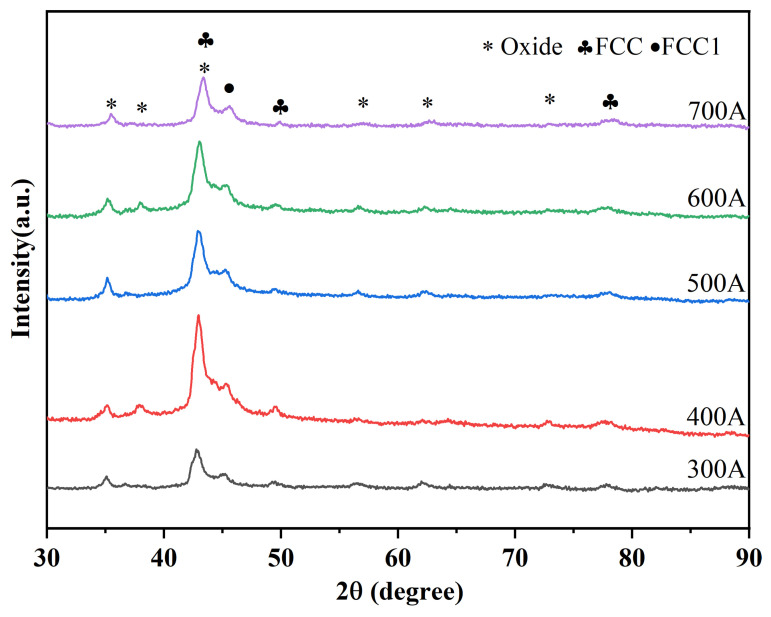
The XRD patterns of CoCrFeNiMo coatings under different spraying currents.

Figure 2 shows the XRD patterns of CoCrFeNiMo coatings prepared by APS under different spraying currents. The CoCrFeNiMo coatings are mainly composed of FCC solid solution phase, FCC1 phase, and oxide phase. Moreover, comparative analysis reveals that changes in spraying current do not cause changes in the phase composition of the coatings. There is a significant phase change between the CoCrFeNiMo powder (Figure 1b) and its corresponding coating. The σ phase in the coating disappears completely, with the FCC phase serving as the main phase. The disappearance of the σ phase is due to the fact that during the spraying process, the powder is instantly heated to a temperature exceeding the stability range of the σ phase, causing the σ phase to decompose into an FCC solid solution [33]. The σ phase is only stable in the medium-low temperature range of 600~800 °C, while the high-temperature environment during spraying far exceeds the upper limit of its stable temperature, leading to the complete decomposition of the original σ phase in the powder [34]. These oxides are mainly composite spinel oxides of the AB_2_O_4_ type. Among the alloying elements of several high-entropy alloys, Cr has the strongest oxygen affinity; therefore, the spinel oxides are most likely composite oxides, such as (Fe,Co,Ni)Cr_2_O_4_ [35].

The FCC diffraction peak with the highest intensity was selected, and the Scherrer equation was used to calculate the grain sizes of the high-entropy alloy coatings under different currents, which were 13.071 Å, 12.652 Å, 12.268 Å, 12.371 Å, and 12.544 Å, respectively. As the current increased, the coating grain size first increased and then decreased. This is because under low-current conditions, the melting degree of the spray powder was insufficient. With the increase in the current, the input energy rose, the cooling area expanded, and the grains were refined. When the spray current further increased, the higher heat input promoted the secondary growth of some grains in the deposited coating. Nevertheless, the multi-component characteristic of the high-entropy alloy inhibited the excessive growth of grains, resulting in only a slight change in the coating grains as the current increased.

**Figure 3:**
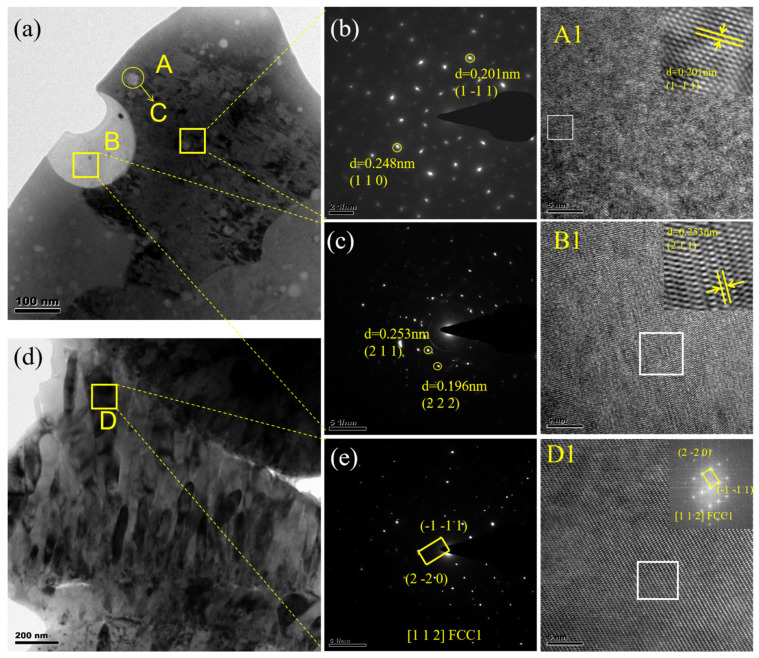
TEM Images of the CoCrFeNiMo Coatings Prepared at a Spraying Current of 500 A: Morphology Images (**a**,**d**), Electron Diffraction Patterns (**b**,**c**,**e**), High-Resolution Images and Fourier Transform Images (**A1**,**B1**,**D1**).

Based on XRD results, it was observed that variations in current exerted minimal influence on the phase composition of the CoCrFeNiMo coatings. Therefore, TEM analysis was conducted exclusively on the coating deposited at a spray current of 500 A. Figure 3 presents the TEM results for the high-entropy alloy coating deposited at 500 A. Figure 3a shows the bright-field TEM image of the high-entropy alloy coating, which primarily consists of light and dark phases. Spherical particles with diameters less than 30 nm are dispersed within the dark gray phase. Select area electron diffraction (SAED) analysis of the corresponding region indicates that the dark gray phase in the coating is the FCC phase (Figure 3b), with a corresponding crystal plane (1 1 0) and (1 −1 1). HR-TEM images of region A and their IFFT transformations further confirm this area as the FCC phase region. EDS analysis of the spherical particles in region C of Figure 3a (Table 1) indicates these particles are primarily Cr-O compounds. These nanospheres serve as critical strengthening phases within the matrix, significantly enhancing the mechanical properties and overall performance of the coating [36]. SAED and HRTEM analyses were performed on the bright phase in Figure 3a. Figure 3c displays the SAED pattern of this bright phase. Calibration of the diffraction spots reveals they correspond to the (211) and (222) crystal plane of the FeCr_2_O_4_ spinel-structured oxide. To further verify this phase, HRTEM observations were conducted on the region, with the results shown in Figure 3(B1). The image displays distinct atomic lattice fringes. Measured using DigitalMicrograph software (Version 3.5), the interplanar spacing was 0.253 nm. This value corresponds to the theoretical interplanar spacing of the (211) plane in the FeCr_2_O_4_ spinel phase. The formation of this spinel oxide primarily resulted from the vigorous reaction between molten alloy droplets and residual oxygen in the environment during the plasma spraying process. Among the alloying elements, both Fe and Cr exhibit high affinity for oxygen. Under rapid cooling and non-equilibrium conditions, they co-precipitate to form thermodynamically stable compound oxides. Spinel-type oxides (AB_2_O_4_) are common protective oxidation products generated during high-temperature oxidation processes.

Figure 3d shows the bright-field phase in another region, exhibiting elongated structures. SAED analysis reveals that this region corresponds to a different FCC phase distinct from Region A. The diffraction spots primarily align with the (−1 −1 1) and (−2 −2 0) crystal plane characteristic of the FCC phase. This diffraction pattern exhibits typical [1 1 2] crystal zone axis orientation. High-resolution Fourier transform analysis of this region confirms that the interplanar spacing and orientation of the bright spots match the SAED results. EDS analysis indicates this area primarily consists of a highly ductile, Cr-rich oxide phase [37].

**Figure 4:**
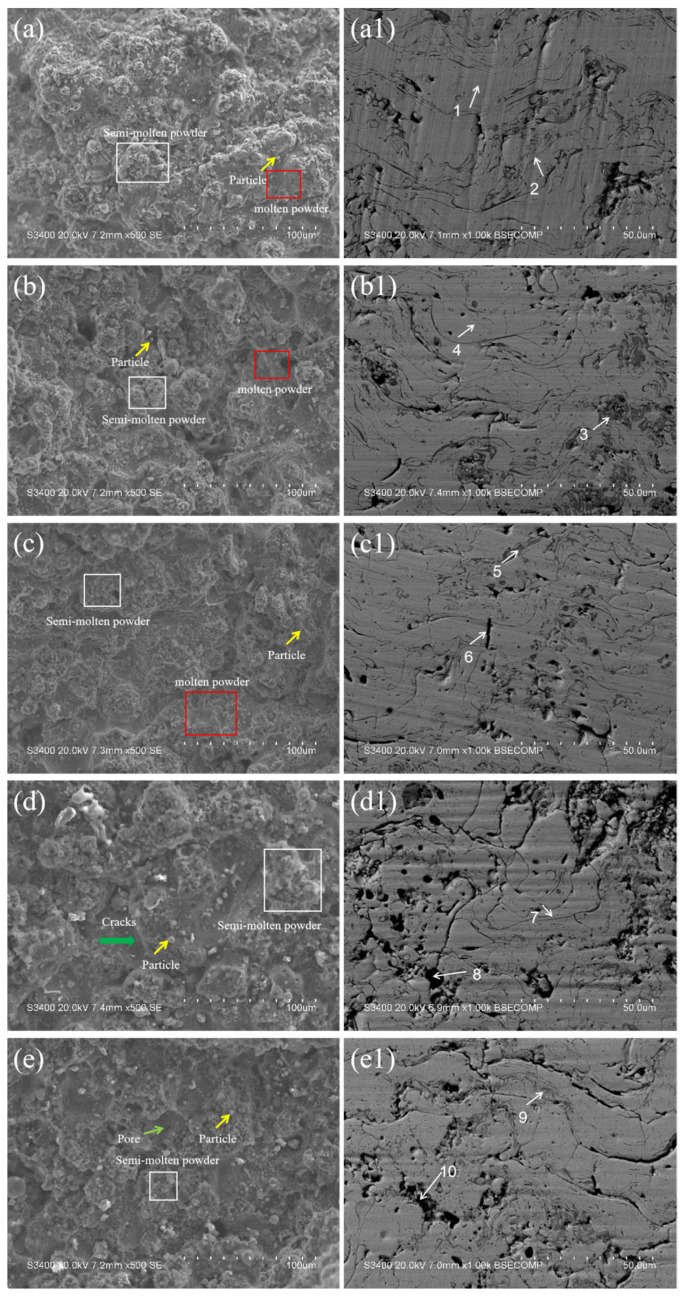
Surface morphology and cross-sectional morphology of CoCrFeNiMo coatings: (**a**,**a1**) 300 A, (**b**,**b1**) 400 A, (**c**,**c1**) 500 A, (**d**,**d1**) 600 A, (**e**,**e1**) 700 A.

Figure 4 presents the surface morphologies (a–e) and cross-sectional morphologies (a1–e1) of the CoCrFeNiMo coatings prepared by APS. As observed from the surface morphologies (a–e), the coating surface is mainly composed of three parts: smooth fully molten regions, stacked semi-molten regions, and unmelted individual powder particles. The particles in the fully molten regions acquire the most energy during the spraying process. These particles are completely melted, and when they impact the substrate surface, they fully spread, flow, and solidify rapidly, forming a typical lamellar structure. Some particles only melt on the surface while remaining unmelted inside during spraying; these semi-molten particles stack together. In addition, there are a small number of unmelted individual powder particles in the coating. These powders retain their original morphology and are embedded in the coating through mechanical interlocking. From the observation of the coating surface morphology, it can be seen that when the spraying current is lower than 500 A, as the spraying current increases, the energy of the plasma flame flow increases, the fully molten region of the coating expands, and the density of the coatings improves. However, when the spraying current exceeds 500 A, loose structures and some pores appear on the coating surface. This is attributed to the splashing of high-entropy alloy powders caused by over-melting. Specifically, the excessively high energy input results in over-melted particles that cannot spread stably when impacting the substrate surface; instead, they splash. This not only reduces the coating deposition efficiency but also forms irregular holes and pores on the coating surface. It can be concluded from the surface morphologies that the coating exhibits the optimal surface morphology when the spraying current is 500 A.

Figure 4(a1–e1) show the BSE cross-sectional morphologies of the coatings under different spraying currents. The figures show that the CoCrFeNiMo coatings exhibit unique microstructures of atmospheric plasma spraying under different spraying currents. The curved lamellar structure of the coating is formed by molten or semi-molten powders, which impact the substrate surface at high speed, spread rapidly, and solidify into lamellae. These lamellae are stacked alternately upward along the deposition direction, forming the main layered structure of the coating. There are elongated dark regions at the boundaries of the lamellar structure. According to the energy spectrum results, these regions are rich in Cr, Fe, and O elements (Table 2 (point 2, 3, 5, 7, 9)). Combined with the XRD and TEM results, it is comprehensively determined that these dark regions are spinel oxide. This is because molten metal droplets are exposed to the atmosphere, and active elements react with oxygen to form oxides. These oxides are driven into the coating by the impact of subsequent molten droplets and are distributed along the lamellar boundaries. In addition, there are some black spherical micropores in the coating cross-section. This is due to the failure of supersaturated gases to escape in time during the spraying process, and the rapid cooling of molten droplets leads to the formation of pores [38].

**Figure 5:**
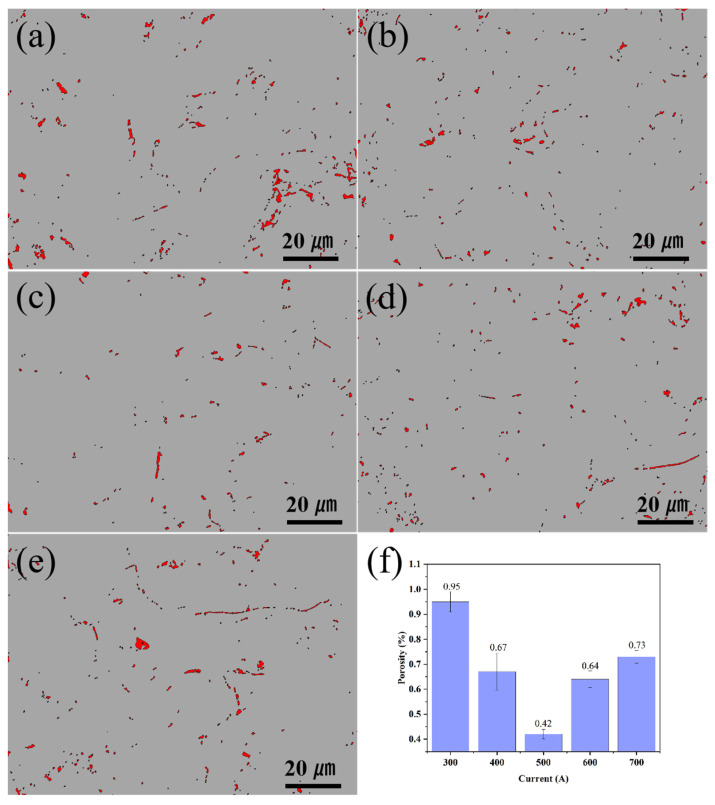
The binarized images and statistical charts of the cross-sectional porosity of the CoCrFeNiMo coatings: (**a**) 300 A, (**b**) 400 A, (**c**) 500 A, (**d**) 600 A, (**e**) 700 A,(**f**) statistical charts.

Figure 5 shows the binarized images and statistical charts of the cross-sectional porosity of ion-sprayed CoCrFeNiMo coatings under different spraying currents. It can be seen from the figure that the spraying current has a significant impact on the densification degree of the coating. When the spraying current is 300 A, the coating porosity is 0.95%. As the spraying current increases, the coating porosity decreases, and when the spraying current reaches 500 A, the coating porosity drops to 0.42%. However, when the spraying current is further increased, the coating porosity does not decrease further; instead, it increases with the increase in spraying current. When the spraying current is 700 A, the coating porosity rises to 0.73%. When the spraying current is lower than 500 A, the reduction in coating porosity is mainly due to the difference in the melting state of the spray powder. At lower currents, the enthalpy and kinetic energy of the plasma jet are insufficient, resulting in incomplete melting of some powders. These molten or semi-molten particles are present in the coating in the form of mechanical interlocking, and irregular pores are generated during the stacking process. Therefore, the coating porosity is relatively high during low-current spraying. With the increase in spraying current, the spray powder obtains sufficient thermal energy and kinetic energy, leading to better powder melting degree and better spreading ability on the substrate surface, thus reducing the coating porosity. When the spraying current exceeds 500 A, the excessive heat input causes the powder to over-melt or even vaporize, resulting in a decrease in the viscosity of the droplets and splashing, thereby forming pores. On the other hand, when the sprayed ions gain excessive energy, residual stress accumulates in the coating. When the residual stress exceeds the critical value, microcracks occur in the coating, which leads to an increase in coating porosity to 0.64% and 0.73% when the spraying current is 600 A and 700 A, respectively.

**Figure 6:**
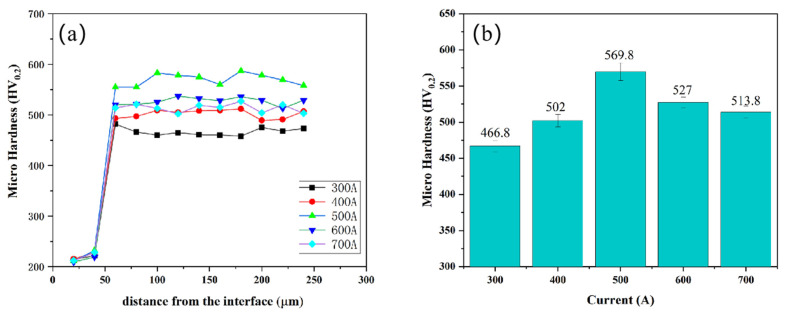
The microhardness changes along the cross-section (**a**) and average microhardness (**b**) of the CoCrFeNiMo coatings.

Figure 6 shows the hardness distribution across the coating cross-section (a) and average microhardness (b). The results clearly indicate that the hardness of the coating region (466.8~569.8 HV) is significantly higher than that of the substrate. During the plasma spraying process, the high-entropy alloy droplets spread rapidly and solidify on the substrate surface, forming a continuous and dense layered structure. Although the coating is composed of multiple stacked layers, the uniform solid solution of each component and the dispersed distribution of nanoscale oxides, through the synergistic effect of solid solution strengthening and second-phase strengthening, make the overall mechanical properties of the coating tend to be consistent, avoiding hardness fluctuations caused by compositional segregation or structural defects. As can be seen from the figure, the variation in coating hardness shows a positive correlation with the variation in coating porosity. With the increase in spraying current, the microhardness of the coating first increases and then decreases. When the spraying current is 300 A, the microhardness of the coating is 466.8 HV; when the spraying current rises to 500 A, the microhardness of the coating increases to 569.8 HV. With a further increase in spraying current, the hardness of the coating begins to decrease, and at 700 A, the coating hardness drops to 513.8 HV. When the current is lower than 500 A, the coating hardness increases with the increase in current. This is attributed to two reasons: on the one hand, as the current increases, the melting state of the powder improves, the droplets spread fully, and interlayer defects and pores are reduced. On the other hand, there are many slender high-hardness metal oxide structures in the coating, and these fine oxide structures are dispersed in the coating and play a certain strengthening role, improving the hardness of the coating. However, when the current exceeds 500 A, the oxidation reaction of the elements in the coating intensifies, and the content of oxides in the coating increases. Moreover, these excessive oxides no longer exist in a slender form but appear as a network in pieces, which in turn reduces the metallurgical bonding strength between the coating layers. During testing, microcracks and uncoordinated plastic deformation occurs, resulting in a decrease in the microhardness of the coating.

**Figure 7:**
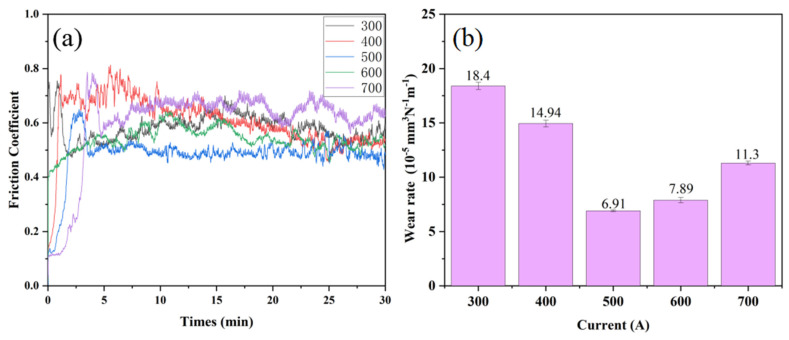
(**a**) friction coefficient curve, (**b**) wear rate chart of the CoCrFeNiMo coatings.

The ability of the material to resist surface wear determines its service life under dynamic contact conditions and is a core indicator for evaluating material reliability. In this experiment, the wear resistance of the CoCrFeNiMo coating was studied at a room temperature of 25 °C, using a GCr15 ball with a diameter of 3 cm as the friction pair. Figure 7a shows the friction coefficients of CoCrFeNiMo coatings prepared under different current intensities. It can be seen from the figure that as the current intensity increases, the friction coefficient of the CoCrFeNiMo coating first decreases and then increases. All coating friction coefficient curves are similar, featuring a rapid running-in stage and a stable stage. Initially, the friction ball begins to adapt to friction, leading to significant fluctuations in the friction coefficient; after a certain period, the frictional fit between the friction ball and the substrate reaches a good level, and the friction coefficient decreases and stabilizes. The average friction coefficients of the different CoCrFeNiMo coatings are 0.59, 0.57, 0.49, 0.55, and 0.65, respectively. Figure 7b presents the variation trend of the wear rate of CoCrFeNiMo high-entropy alloy coatings prepared under different spraying currents. It can be clearly observed from the figure that the wear rate of the coating first decreases and then increases, which is consistent with the trend of coating hardness. When the spraying current increases from 300 A to 500 A, the wear rate of the coating decreases from 18.4 × 10^−5^ mm^3^/(N·m) to 6.91 × 10^−5^ mm^3^/(N·m); when the current further increases to 700 A, the coating wear rate rises to 11.3 × 10^−5^ mm^3^/(N·m).

**Figure 8:**
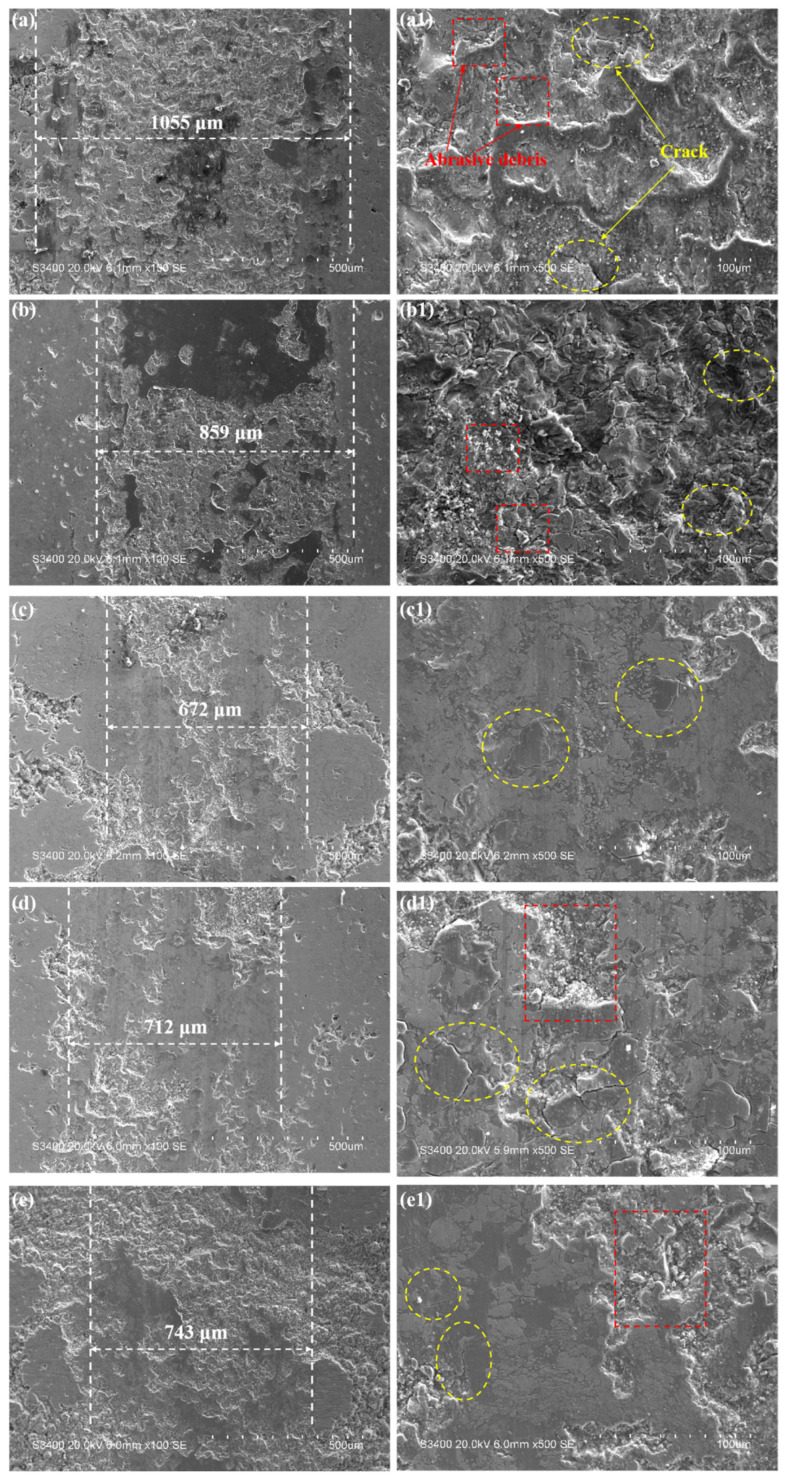
The room temperature friction and wear images of the CoCrFeNiMo coatings: (**a**,**a1**) 300 A, (**b**,**b1**) 400 A, (**c**,**c1**) 500 A, (**d**,**d1**) 600 A, (**e**,**e1**) 700 A.

To further investigate the reasons for the variation in the friction coefficient of the coatings, this study analyzed the micromorphology of the coating after friction and wear, as shown in Figure 8. It can be seen from the figure that the wear scar width is consistent with the trend of the friction coefficient. The wear scar widths of the different CoCrFeNiMo coatings are 1055 μm, 859 μm, 672 μm, 712 μm, and 743 μm, respectively, which show a positive correlation with their corresponding friction coefficients. Coatings prepared by different processes exhibit significant differences in wear morphology. Among them, the coating prepared with the medium current intensity shows the most excellent surface condition. The wear track is flat and smooth, and the cracks generated on its surface are also small. This wear morphology characteristic is completely consistent with its low friction coefficient performance. In contrast, coatings prepared with other process parameters show varying degrees of surface damage. The coating prepared at low current had a large number of abrasive particles and obvious plastic deformation characteristics on its surface, indicating that severe abrasive wear and extrusion deformation occurred in the material during the friction process. Although the extrusion deformation of the coating prepared at a high current was reduced, more abrasive particles and microcracks appeared, which is also unfavorable for the improvement of friction performance. In summary, the influence of process parameters on coating performance is mainly achieved by changing the microstructure of the materials. A relatively low current intensity leads to insufficient coating density, making it difficult to form a complete protective layer; while an excessively high current intensity causes grain coarsening and residual stress accumulation, reducing the overall performance of the material.

**Figure 9:**
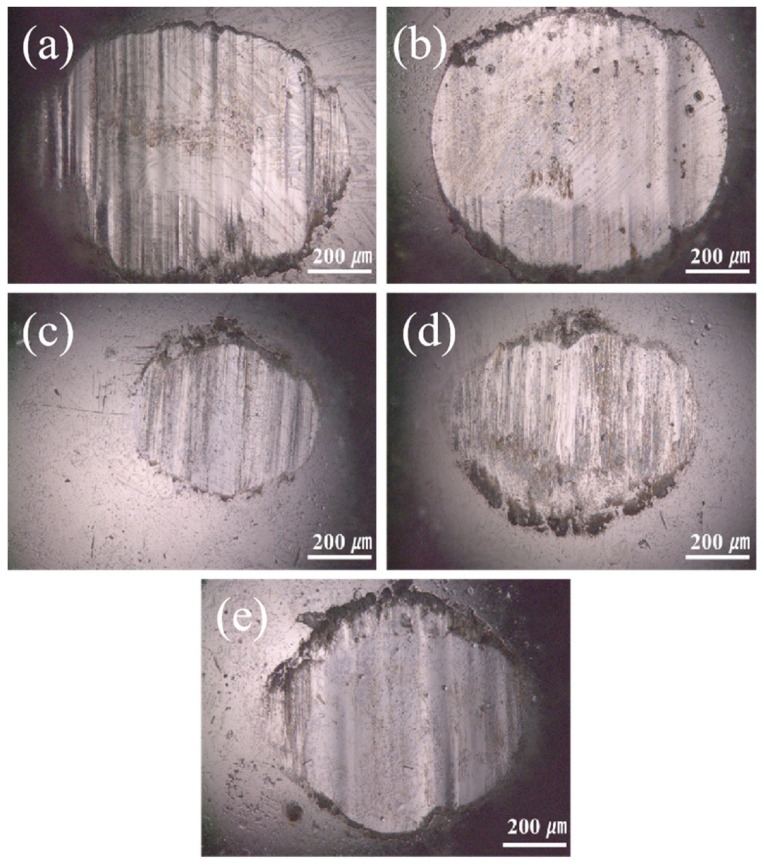
Wear of the GCr15 balls sliding against the CoCrFeNiMo coatings: (**a**) 300 A, (**b**) 400 A, (**c**) 500 A, (**d**) 600 A, (**e**) 700 A.

Figure 9 shows the wear morphology of the GCr15 counterface ball surface after sliding against the coating. As observed from Figure 9, the wear area of the counterface ball is much larger than the wear scar on the coating surface. There are furrows along the sliding friction direction on the counterface ball surface, indicating that abrasive wear occurs during the wear process; in addition, a certain amount of dark Fe_2_O_3_ is present at the edges of the wear scars. Moreover, with the change in current, the variation trend of the wear degree of the counterface ball is consistent with that of the coating [39].

The friction and wear resistance of the plasma-sprayed CoCrFeNiMo coatings are consistent with the variation trends in the hardness and cross-sectional porosity. When the spraying current is lower than 500 A, the wear resistance of the coating enhances as the current increases. This is mainly attributed to the fact that a moderate current input provides optimal energy, which promotes the full melting and spreading of powder particles, thereby effectively reducing the coating porosity and improving the density and bonding strength with the substrate of the coatings. According to XRD and TEM analyses, as the spray current increases, the grain size of the coating first increases and then decreases. At a current of 500 A, the coating exhibits the smallest grain size, indicating that the variation in current plays a role in grain refinement. This effect works synergistically with the dispersed oxide phases in the coating to jointly enhance the wear resistance of the coatings. The matrix phase of the coating is mainly the FCC phase, in which nano-spherical particles are dispersed. These spherical particles play an important role in the dispersion strengthening of the coating and can effectively resist plastic deformation during the wear process [40,41]. The coating contains a certain amount of FeCr_2_O_4_ spinel oxide, which has high hardness and also plays a supporting role in the coating [42]. However, when the spraying current is further increased to 700 A, the coating’s hardness begins to decrease, the porosity increases, and the wear resistance of the coating deteriorates accordingly. This is because an excessively high current input may cause overheating of the molten pool, leading to excessive grain growth and the formation of coarse structures, thereby reducing the overall strength and toughness of the coating. In addition, excessive heat input may also intensify oxidation reactions, leading to the formation of harmful oxides or the decomposition/coarsening of original hard phases, weakening their strengthening effect. At the same time, excessive thermal stress may generate more microcracks and defects inside the coating, making the coating more prone to fatigue spallation and brittle fracture during friction, thereby accelerating wear [32,43]. Based on comprehensive analysis, when the spraying current is 500 A, the coating exhibits the highest hardness and the best wear resistance.

## 4. Conclusions

In this study, a series of CoCrFeNiMo coatings were prepared by adjusting the spraying current, and the main conclusions are as follows:

(1) The spraying current has little effect on the phase composition of the coatings. All coatings are mainly composed of the FCC phase, FeCr_2_O_4_ spinel phase, and Cr-rich oxide phase. A notable feature of the coating microstructure is the formation of spherical Cr oxide particles with a diameter of less than 30 nm in the FCC matrix; these nanoparticles play a key role in the dispersion strengthening of the coatings.

(2) The spraying current has a significant impact on the compactness and mechanical properties of the coatings. Both porosity and microhardness first improve and then deteriorate with increases in current. Under the optimized current of 500 A, the coating achieves the lowest porosity (0.42%) and the highest microhardness (569.8 HV).

(3) The wear resistance of the coatings is consistent with the variation laws of microstructure and mechanical properties, and also reaches the best state at 500 A. At this current, the wear rate is the lowest (6.91 × 10^−5^ mm^3^/(N·m)) and the stable friction coefficient is the lowest (0.49). According to the analysis of coating wear scars, coatings prepared at low current suffer from severe abrasive wear and plastic deformation due to insufficient hardness, while coatings prepared at high current show reduced deformation, but more abrasive particles and microcracks are generated in them due to excessive oxidation and increased brittle phases, which also deteriorates the wear resistance.

## Figures and Tables

**Table 1:** Elemental distribution (at.%) in microregions (Figure 3).

Regions	O	Co	Cr	Fe	Ni	Mo
A	0	20.1	15.9	18.2	20.1	25.5
B	58	2.2	28.4	8.1	1.2	2.0
C	58.2	0.8	36.8	1.4	0.6	2.3
D	38.7	6.7	32.6	5.2	6.1	10.7

**Table 2:** Elemental distribution (at.%) in microregions (Figure 4).

Regions	O	Co	Cr	Fe	Ni	Mo
1	7.54	19.86	17.49	18.7	18.38	18.01
2	48.65	8.38	16.56	10.6	7.36	8.45
3	48.15	8.53	16.1	12.91	6.1	8.22
4	11.14	17.56	18.38	17.98	17.31	17.64
5	49.34	8.31	16.82	12.19	5.62	7.66
6	21.27	15.15	13.64	21.43	14.86	13.66
7	42.3	8.62	18.68	12.19	9.31	8.91
8	12.18	18.26	14.88	17.69	16.91	20.07
9	41.37	8.85	17.59	13.46	9.08	9.65
10	10.09	18.32	15.88	18.32	17.66	19.73

## Data Availability

Data are contained within the article.
